# Evaluation of a Post-Operative Therapy Protocol after Epithelium-Off Corneal Cross-Linking in Patients Affected by Keratoconus

**DOI:** 10.3390/jcm11237093

**Published:** 2022-11-30

**Authors:** Karl Anders Knutsson, Paola Noemi Genovese, Giorgio Paganoni, Oriella Ambrosio, Giulio Ferrari, Arianna Zennato, Madeleine Cataldo, Michela Caccia, Paolo Rama

**Affiliations:** Cornea and Ocular Surface Unit, San Raffaele Scientific Institute, 20132 Milan, Italy

**Keywords:** corneal collagen cross-linking, keratoconus, long-term cross-linking, cross-linking, corneal ectasia

## Abstract

A large retrospective study evaluated the safety of a post-operative therapy protocol after epithelium-off corneal collagen cross-linking (CXL). In total, 1703 eyes of the 1190 patients with progressive keratoconus were enrolled in a retrospective cohort study in a tertiary care university hospital. CXL was performed using a standardized technique (Dresden protocol: 0.1% riboflavin solution containing dextran 20% for 30 min during the soaking phase followed by 30-min ultraviolet A irradiation (3 mW/cm^2^)). Postoperatively, a bandage contact lens was applied, and therapy included a topical fluoroquinolone antibiotic until the epithelium healed, followed by topical fluorometholone treatment for three weeks. Post-operative complications were recorded and analyzed. No cases of infectious keratitis occurred, whereas peripheral sterile infiltrates were observed in 1.17% of cases. Trace haze was typically present but did not have an impact on visual acuity. In fifteen cases (0.88%), visually significant anterior stromal opacity developed. Mild signs of dry eye were observed in 22 eyes (1.29%). The present study demonstrates that a post-operative treatment protocol including fluoroquinolone antibiotics and a BCL in the first phase until complete epithelial healing, followed by a three-week period of topical steroid treatment is safe and not associated with the development of microbial keratitis.

## 1. Introduction

Keratoconus is a noninflammatory degenerative ectasia of the cornea, which is typically bilateral and asymmetric and often progresses over time [1]. In the initial stages, keratoconus determines reduction of vision due to increased myopia and irregular astigmatism. Spectacles provide adequate visual rehabilitation in early stages of disease, until irregular astigmatism increases, requiring correction with rigid contact lenses to achieve visual rehabilitation. In patients with progressive keratoconus, visual acuity may decrease due to corneal opacification and corneal transplantation may become necessary. Numerous studies have confirmed the effectiveness of CXL in halting the progression of keratectasia in both the adult [2,3,4,5,6,7,8,9] and pediatric [1,10,11,12,13,14,15,16,17,18] populations. In recent years, long-term results of CXL in patients with over ten-years of follow-up have been reported [19,20,21].

Although there are alternative techniques for performing CXL without removing the epithelium (epithelium on CXL or trans-epithelial), CXL associated with removal of the epithelium (epithelium-off) represents the current gold-standard treatment [8,22]. Removal of the epithelium, however, is associated with a significant risk of corneal infection, since this represents removal of one of the main physical barriers against microbes and other pathogens [23,24]. Different risk factors have been identified including, epithelial defects, use of a bandage contact lens (BCL), atopy, diabetes and topical steroids [23,24,25]. Only few monocentric studies with large patient populations have focused specifically on post-operative infection and safety outcomes [26,27,28,29]. The present study aims to add substantial information to this subject area and specifically evaluates the safety of a postoperative protocol used for a large cohort of patients.

## 2. Materials and Methods

In this retrospective study, we analyzed the records of 1703 eyes of 1190 consecutive patients with progressive keratoconus from June-2008 to June 2021. Local ethics committee approval (San Raffaele Scientific Institute Ethical Committee) was achieved and informed consent was obtained by all patients or their parents in case of minors. The study adhered to the tenets of the Declaration of Helsinki.

Keratoconus was diagnosed using a combination of slit-lamp findings and topography/tomography examinations which revealed typical keratoconus patterns associated with altered parameters such as maximum keratometry (Kmax), central keratometry and inferior–superior asymmetry [30]. Disease progression was defined as an increase of Kmax by at least 1 Diopter(D) in one year. All patients included in the study had at least one year of follow-up. Patients with other forms of ectasia were not included in the study. Patients with minimum corneal thickness <400 µm, corneal scarring, history of infectious keratitis, mental retardation, dry eye, corneal endothelial cell pathology, autoimmune disease or previous ocular interventions were excluded. Patients habitually using contact lenses were instructed to remove them three-weeks prior to examinations. Statistical analysis was performed with SPSS (Version 20.0, IBM Corp., Armonk, NY, USA). The non-parametric Kruskal–Wallis test was utilized to compare complications between eyes receiving different topical antibiotics.

### 2.1. Surgical Technique

CXL with riboflavin and Ultra-Violet A (UVA) light was performed on all patients following a standard protocol [8]. Topical anesthetics (lidocaine 4% and oxybuprocaine hydrochloride 0.2%) were applied 15-min prior to the procedure. The central 9 mm diameter of corneal epithelium was removed with a blunt spatula and 0.1%riboflavin-20%dextran solution (Ricrolin; Sooft, Montegiorgio, Italy) was applied for 30 min during the soaking phase. The central cornea (9 mm beam diameter) was then exposed to UVA radiation (370 nm) through a solid-state device (Vega X-Linker; CSO, Firenze, Italy) at 3 mW/cm^2^ for 30 min (5.4 J/cm^2^ total energy) with instillation of riboflavin solution every 2.5 min. Centration of the treatment area was controlled by the surgeon and the patient maintained fixation on a target light. Topical lidocaine (4%) was instilled during the procedure for a maximum of two times to prevent riboflavin dilution. All patients successfully underwent surgery. For some pediatric patients, a parent was allowed in the operating theater in order to reduce potential anxiety. Most patients received topical anesthesia alone. Postoperatively, topical levofloxacin 0.5% was instilled and a bandage contact lens (Purevision; Bausch & Lomb, Rochester, NY, USA) was applied for 5 days until complete re-epithelialization was achieved. 

### 2.2. Postoperative Regimen

Patients received topical 0.5% levofloxacin (*n* = 900, 52.8%) or 0.3% ofloxacin (*n* = 803, 47.2%) 6-times daily for 5 days, sodium hyaluronate lubricant drops 0.15% four times daily for 30 days, and topical fluorometholone 0.1% 2-times daily for 21 days after bandage contact lens removal.

Patients were examined post-operatively one day after treatment, at five days for bandage contact lens removal, at one month, six months, one year, and once yearly for the subsequent follow-up examinations. 

## 3. Results

In total, 1,703 eyes of 1190 patients were selected for this retrospective study. Mean age of the patients was 22.88 ± 5.82 years, 901 subjects were male and 289 female (M:F ratio 3.1:1), and 179 patients (15.0 %) were aged under 18 with an adult/minor ratio of 5.65. Average pre-operative pachymetry of the thinnest point was 476.87 ± 33.11 µm; all patients had a corneal thickness greater than 400 µm. In 12 eyes, the procedure was performed under general anesthesia, while in the remaining cases, topical anesthesia alone was utilized. Table 1 summarizes the demographic and clinical characteristics of our cohort.

With regards to postoperative infections, surprisingly no individuals developed infectious keratitis. Twenty patients (1.17%) were diagnosed with peripheral corneal infiltrates at the one-week postoperative examination. Infiltrates resolved after bandage contact lens removal and appropriate management. In these patients, the BCL was removed and cultured for possible microorganisms, with negative results in all cases. Topical fluoroquinolone antibiotic was prolonged at the same dose until a negative culture was confirmed (usually 4 days after BCL removal), and then topical steroids were applied for three weeks as the original protocol, with full recovery in all cases and no evolution towards infectious keratitis. One patient presented with a peripheral corneal infiltrate seven months after CXL which was successfully treated with a short course of antibiotics and steroids. The etiology was likely an immune infiltrate related to blepharitis. In three atopic patients, epithelial irregularity and punctate keratopathy developed due to ocular surface inflammation and improved with an increase in lubricant dosage. A very mild form of corneal trace haze was present in all cases but resolved from 6 to 12 months after treatment and typically did not impact visual acuity, except in fifteen cases in which anterior stromal opacity developed (0.88%). Mild signs of dry eye presenting with sectorial punctate keratitis were present in 22 eyes (1.29%) immediately after contact lens removal. All cases resolved after appropriate management with lubricants. No cases of persistent epithelial defects were encountered. No significant differences in complications were revealed when comparing the type of antibiotic used (Ofloxacin vs. Levofloxacin). Table 2 summarizes the major complications in our cohort.

## 4. Discussion

CXL has transformed the management of keratoconus and has been proven effective in both the adult and pediatric population. The present study aims to evaluate the safety of a postoperative protocol that includes the use of topical fluoroquinolones and a bandage contact lens, and topical steroids administered only after full epithelial healing.

Previous studies under various contexts have described infectious keratitis as a rare complication, with three large retrospective studies reporting percentages of 0.17% (4/2350 eyes in Turkey), 0.5% (11/2025 eyes in Northern India) and 1.3% (7/532 eyes in Southern India) [26,27,28]. In our cohort we did not observe any cases of infection, possibly due to the temperate climate in our region (compared to India and Turkey) and to the immunosuppressive status of our patients: no patients were receiving chronic topical steroids or oral immunosuppressive therapy. Furthermore, meticulous instructions were given to every patient stressing the problems related to contamination of the operated eye and risk factors related to BCL wear. In the study by Shetty, et al. the four individuals with infectious keratitis had associated risk factors. Two patients were affected by vernal conjunctivitis with previous chronic use of topical steroids, one patient was receiving oral steroids for bronchial asthma and another patient was utilizing oral cyclophosphamide for treating eczema [27]. Maharana et al. reported seven cases of keratitis after accelerated CXL and noted that four (57.1%) of the cases were associated with vernal keratoconjunctivitis [28]. Our patient population had a 7% prevalence of atopy but inflammation was minimal in the peri-operative period, further supporting the absence of postoperative keratitis. Similar to other groups, we reported sterile infiltrates in 20 eyes (1.17%) which resolved after bandage contact lens removal and topical steroid therapy without any consequences or development of infection [27,28,31]. One patient presented with a peripheral corneal infiltrate seven months after CXL which was successfully treated with a short course of antibiotics and steroids. The etiology was likely an immune infiltrate related to blepharitis and not related to CXL. 

A recent study of a large cohort, divided patients into two groups, those treated postoperatively with a BCL, topical antimicrobial and steroids (Group 1) and those who received only a topical antimicrobial until healing of the epithelial defect before introduction of topical steroids (Group 2) [29]. Staphylococcus aureus keratitis occurred in 9 eyes (0.71%) of the first group only and the authors conclude that using a BCL and steroids before epithelial healing were associated with microbial keratitis. However, they were unable to determine whether the use of BCL, topical steroids or their combination has led to the increase in the incidence of microbial keratitis. Since we had no cases of infections in our cohort despite all patients had postoperative BCL application, we may speculate that postoperative BCL use may have a less significant impact compared to the use of topical steroids before full epithelial healing. However, we must also consider that adequate patient education may have a crucial role in preventing contact lens related infectious keratitis. Furthermore, the risk of infection in our population could be lower due to the reduced presence of atopy (7% vs. 50.7%). Another factor that must be considered is that in our study, microbial keratitis may be underestimated since peripheral corneal infiltrates were considered sterile after a negative culture on the contact lens, without performing corneal scraping.

The presence of corneal haze differs significantly in different reports, with some authors reporting rates of 9%, 9.1% and 100% [4,7,24,27]. These differences are most likely due to a different evaluation between observers and likely to be underestimated due to their low impact on visual acuity. We reported the presence of very mild trace haze in all patients, which is in line with the prospective study by Wittig-Silva et al. [7]. Trace haze disappeared after 6- to 12-months post-treatment and did not impact on visual acuity results. In total, 15 eyes (0.88%) developed a more severe form of anterior corneal opacity, which interfered with vision. 

Mild signs of dry eye presenting with sectorial punctate keratitis were present in 22 eyes (1.29%) immediately after contact lens removal. All cases were temporary and resolved after topical lubricants were utilized more frequently. In three atopic patients, epithelial irregularity and punctate keratopathy developed due to ocular surface inflammation and also improved with an increase in lubricant dosage. 

The principal weakness of this study is related to its retrospective nature. Large prospective studies are needed to evaluate the potential risk factors associated with the rare event of postoperative microbial keratitis. The impact of precise postoperative instructions and patient compliance is also under evaluated and needs further study. Another aspect that must be addressed is related to the analysis of paired cases. Ideally only one eye should be randomly selected for analysis. However, in this specific study, we deliberately decided to use data from both eyes to avoid any data loss, considering that postoperative infection is a rare event. This approach can however lead to skewed data. Furthermore, not all patients received the same antibiotic drug, since approximately half of the population utilized levofloxacin and the other half ofloxacin. This difference is unlikely to bear a clinical impact and resembles “real-life” situations where drug shortages may occur and patients may use similar antibiotics of the same class but not identical to those prescribed by their ophthalmologist. No significant differences in complications were found between eyes receiving a different topical antibiotic.

In conclusion, the results from this large retrospective cohort study confirm the safety of a postoperative treatment protocol that includes use of fluoroquinolone antibiotics and a BCL in the first phase until complete epithelial healing, followed by a three-week treatment period with topical steroids.

## Figures and Tables

**Table 1:** Demographic and clinical characteristics of patients undergoing collagen cross-linking.

Number of Patients	1703
Age (years)	22.88 ± 5.82
Male:female ratio	3.1
Adult:minor ratio	5.65
Preoperative minimum corneal thickness (µm)	476.87 ± 33.11.
Maximum keratometry (diopters)	55.88 ± 5.92
Atopy (%)	120/1703 (7.0%)
Race (*n* = number of eyes, %)	Caucasian (*n* = 1683, 98.8%) Latin American (*n* = 15, 0.88%) Middle-Eastern (*n* = 3, 0.18%) Asian (*n* = 2, 0.12%)

**Table 2:** Postoperative complications of Corneal Cross-Linking.

Complication	N (Percentage %)	Ofloxacin vs. Levofloxacin (*p*-Value)
Mild trace haze	1703 (100%)	Not applicable
Temporary dry eye	22 (1.29%)	(*n* = 10) vs. (*n* = 12) (*p* = 0.624)
Sterile peripheral infiltrates	20 (1.17%)	(*n* = 11) vs. (*n* = 9) (*p* = 0.456)
Epithelial irregularity	3 (0.18 %)	(*n* = 1) vs. (*n* = 2) (*p* = 0.564)
Inflammatory infiltrate	1 (0.06 %)	(*n* = 1) vs. (*n* = 0) (*p* = 0.332)

## Data Availability

The data related to this study are not publicly available due to privacy concerns.
